# Natural Products for the Control of *Scaphoideus titanus* in Vineyards: A Summary of Five-Year Field Trials

**DOI:** 10.3390/insects17010083

**Published:** 2026-01-10

**Authors:** Stefan Cristian Prazaru, Luigi Forlin, Leonardo Cera, Lisa D’Ambrogio, Alberto Pozzebon, Carlo Duso

**Affiliations:** 1 Department of Agronomy, Food, Natural Resources, Animals and Environment, University of Padova, Viale dell’Università 16, 32050 Legnaro, Padova, Italy; leonardo.cera@unipd.it (L.C.); lisa.dambrogio@unipd.it (L.D.); alberto.pozzebon@unipd.it (A.P.); 2 Centro per l’Educazione la Cooperazione e l’Assistenza Tecnica (CECAT), Via della Borsa, 1/a, 31033 Castelfranco Veneto, Treviso, Italy; lforlin70@gmail.com

**Keywords:** Flavescence dorée, organic management, *Scaphoideus titanus*, vector, viticulture

## Abstract

*Scaphoideus titanus* Ball, 1932 (Hemiptera: Cicadellidae), the main vector of the phytoplasma (FDp) causing Flavescence dorée, is considered a challenging problem in European vineyards. Natural insecticides permitted for *S. titanus* control in Italian organic viticulture are generally considered of low-to-moderate efficacy. Morever, it should be mentioned that their optimal use requires a careful approach when preparing the insecticide solutions. Synthetic insecticides used in Italian viticulture are included in only two IRAC groups, indicating potential risks for resistance. Therefore, there is a need to identify alternatives or control tools complementary to synthetic insecticides. In this study, we summarize the results obtained in five field experiments (2021–2025) using the main alternatives to synthetic insecticides (i.e., pyrethrins, kaolin, azadirachtin, *Beauveria bassiana*, and potassium salts of fatty acids). In all seasons, pre-insecticide application populations did not differ significantly among treatments, whereas differences emerged after insecticide applications. The overall results showed that pyrethrin-based insecticides were the most effective in reducing *S. titanus* nymph densities. Kaolin provided intermediate but comparatively stable levels of control across the years, whereas *B. bassiana* showed moderate yet highly variable efficacy. The findings obtained here can be considered in designing management strategies for *S. titanus* in organic viticulture that require an integrated approach combining pyrethrins with complementary, lower-impact tools to achieve effective and sustainable control over time. At the same time, natural insecticides can be incorporated into IPM strategies in conventional vineyards to prevent resistance.

## Simple Summary

*Scaphoideus titanus,* vector of the Flavescence dorée phytoplasma (FDp), is difficult to control in organic vineyards where permitted natural insecticides often show limited efficacy and require careful spray preparation. Because synthetic insecticides used in Italy belong mainly to only two IRAC groups, resistance risk increases and alternative/complementary tools are needed. Across five field trials (2021–2025) testing pyrethrins, kaolin, azadirachtin, *Beauveria bassiana*, and potassium soap, pre-treatment nymph densities were similar among treatments, but differences appeared after spraying. Pyrethrins were consistently the most effective. Kaolin provided intermediate but stable control across the years, while *B. bassiana* showed moderate and highly variable performance. Results support integrated programs in organic viticulture: this involves using pyrethrins as the core tool and combining them with complementary, lower-impact options to improve sustainability over time. These alternatives can also be incorporated into IPM in conventional vineyards to reduce selection pressure and help prevent resistance.

## 1. Introduction

Flavescence dorée (FD) is a grapevine disease associated with ‘Flavescence dorée’ phytoplasma (FDp), which due to its high damaging potential to viticulture is currently considered a priority quarantine disease in Europe [1,2]. FDp can be transmitted by a number of insect species, but the leafhopper *Scaphoideus titanus* Ball, 1932, is widely recognized as its main vector [3,4]. Accidentally introduced into Europe in the 20th century, *S. titanus* rapidly adapted to European vineyards, becoming responsible for the spread of FD epidemics [3,5].

*Scaphoideus titanus* is univoltine: it overwinters as an egg and in spring, hatching begins progressively and may extend for more than a month [3]. Adult emergence occurs from late June to late August, depending on vineyard altitude. Females lay an average of 60 eggs during their lifespan, and the prolonged hatching and adult emergence periods generate a strong overlap of developmental stages (nymphal instars and adults), resulting in prolonged infectious risk throughout the growing season [4,5,6]. This biology, characterized by a long hatching window and an extended adult phase, makes vector control particularly challenging.

Insecticide applications for the control of *S. titanus* are mandatory in Italy and other European countries, such as Switzerland, Austria, and Slovenia, where FD has become established. Chemical control represents the main pillar of FD management strategies together with the rouging of symptomatic vines and the use of healthy planting material [1,2,7]. In organic vineyards, however, vector control relies almost exclusively on natural pyrethrins because the few other insecticides authorized in these systems (e.g., azadirachtin, potassium salts of fatty acids, and entomopathogenic fungi) have generally shown lower and more inconsistent efficacy [8,9,10,11]. It should be mentioned that the results obtained using natural insecticides requires a careful approach in preparing insecticide solutions (e.g., water pH and/or water hardness); the lack of these conditions could compromise their expected efficacy.

Pyrethrins are plant-derived substances belonging to IRAC group 3A sodium channel modulators, like synthetic pyrethroids [12]. Although they provide a rapid knock-down effect, their persistence is extremely limited due to both UV light and temperature-dependent degradation, an issue that makes their performance highly sensitive to spray timing and environmental conditions; on the other hand, synthetic pyrethroids can lose effectiveness under high-temperature conditions [11,13,14,15]. The combination of prolonged egg hatching and low residual activity can require tight applications during the growing season to maintain adequate control [11]. This entails high costs, a concrete risk of selecting resistant populations, and potential negative impacts on beneficial arthropods. Since pyrethrins and pyrethroids share the same mode of action, reduced efficacy of pyrethrins could, in the long term, compromise the performance of synthetic pyrethroids, which remain crucial in integrated systems [12]. This scenario is further exacerbated by the fact that the introduction of new insecticides with innovative modes of action into the market is slowed by rising costs and regulatory constraints [16].

To reduce dependence on pyrethrins in organic viticulture and pyrethroids in conventional viticulture and to mitigate associated risks, research has increasingly focused on alternative products characterized by low impact on human health and the environment. Kaolin, a clay-based mineral used as a particle film, is one of the most promising solutions. It acts through physical and behavioral mechanisms, interfering with visual cues and insect locomotion and creating a barrier that reduces feeding and oviposition [17,18]. Kaolin also helps mitigate thermal stress and lower leaf temperature, with indirect benefits for vine physiology [19,20]. Recent studies have evaluated its efficacy against *Erasmoneura vulnerata* (Fitch, 1851) and *Hebata* (=*Empoasca*) *vitis* (Göthe, 1875) (Hemiptera: Cicadellidae) and *S. titanus*, with variable results [10,18,21,22,23].

Among alternatives, entomopathogenic fungi, particularly *Beauveria bassiana* Vuill, are listed in the UNF Fungal IRAC group. *B. bassiana* acts both through direct contact and through the potential endophytic colonization of plant tissues, which may induce the production of defensive metabolites [24,25]. However, its field efficacy is often limited or inconsistent, being strongly affected by climatic conditions and by the persistence of spores on plant surfaces [26,27].

Azadirachtin, the main limonoid extracted from neem seeds (*Azadirachta indica* Juss), represents another botanical insecticide of interest for pest and vector control. It is considered one of the most successful botanical insecticides used in agriculture worldwide and is widely employed in organic farming as a broad-spectrum biopesticide [28,29]. Azadirachtin is listed in the IRAC group UN because of uncertainties over a unique mode of action. It is considered to act mainly as an antifeedant and has insect growth-disrupting effects mediated by interference with the neuroendocrine regulation of ecdysteroids and juvenile hormones, as well as additional impacts on neural transmission, such as the partial blockage of calcium channels and inhibition of cholinergic excitation [28]. Azadirachtin-based products generally show low mammalian toxicity and a positive ecotoxicological profile, but their high photosensitivity and rapid degradation result in low residual activity on foliage [28,29]. Azadirachtin-based formulations have already been used in viticulture against leafhoppers, including *S. titanus* and other sucking insects with performance strongly influenced by environmental conditions and the timing of applications [8,30,31].

Potassium salts of fatty acids (insecticidal soaps) are another option available in viticulture. These formulations are contact insecticides for the control of soft-bodied arthropods such as aphids and mites, acting mainly by disrupting the waxy cuticle and cell membranes and causing the rapid desiccation of exposed individuals [32]. Laboratory and greenhouse studies on *Myzus persicae* (Sulzer, 1776) (Hemiptera: Aphididae) have shown that insecticidal soaps can induce very high acute mortality when directly sprayed on the insects, with LC_50_ values in the low g L^−1^ range but virtually no residual activity on treated foliage [32,33]. However, their efficacy is strongly dependent on thorough spray coverage and on the position of the target insects within the canopy, and their selectivity towards natural enemies is only partial: some formulations can negatively affect aphid parasitoids and coccinellid larvae under direct exposure while being less harmful to coccinellid adults and to parasitoids developing inside mummies [32,33]. In vineyards, these characteristics imply that potassium salts of fatty acids may contribute to *S. titanus* suppression only as short-lived, contact products [8,11].

Despite growing interest in alternative solutions, multi-year studies conducted under real operational conditions and directly comparing different strategies in organic vineyards are still relatively rare. To date, one of the few long-term studies is the Swiss trial evaluating kaolin against *S. titanus* [10]. This study focuses on a single particle film product and does not cover the range of natural insecticides available in viticulture. Most available research focuses on single-year trials, small experimental plots, or partial evaluations, limiting the ability to draw robust conclusions on the stability of product performance [34].

The present study aimed to compare the efficacy of natural insecticides for the control of *S. titanus*. This study, conducted over five consecutive years (2021–2025), provides multi-year field data to assess the potential and limitations of these alternative strategies and to support the development of more sustainable control approaches that can be incorporated into organic and integrated pest management (IPM) programs against the main vector of FDp.

## 2. Materials and Methods

### 2.1. Experimental Design and Sampling Procedures

The experimental trials were conducted over five consecutive years, from 2021 to 2025, in commercial vineyards located in northeastern Italy (Veneto region) and managed according to certified organic standards. During 2021–2023, experiments were carried out in the province of Treviso (Villorba, Sarmede, and Ponzano), in 2024, experiments were carried out in the province of Padova (Monselice), and in 2025, they were carried out in the province of Venezia (Cona). In all sites, vineyards aged between 11 and 16 years, comprised the Glera variety, and were trained with the Sylvoz system. All farmers routinely applied copper- and sulfur-based fungicides to control grape pathogens.

A randomized block design was adopted, with three replicates per treatment in 2021 and 2023 and four replicates in 2022, 2024, and 2025. Each experimental unit consisted of three-six contiguous rows, 60 to 120 m in length, and all assessments were performed exclusively on the two central rows of the unit to minimize edge effects. The five following products authorized for use in organic agriculture and representing the main modes of action available for pest management in vineyards (Table 1) were tested:

- Pyrethrins (Biopiren^®^, Biogard—CBC Europe, Bergamo, Italy), a contact insecticide based on natural pyrethrins with rapid knock-down activity but limited persistence [12];
- Azadirachtin (Oikos^®^, Sipcam Italia S.p.A, Milan, Italy), based on 26 g/L azadirachtin A, an insecticide affecting molting, feeding, and behavior, active against juvenile stages of leafhoppers [28,29];
- Potassium salts of fatty acids (Flipper^®^, Bayer CropScience S.r.l., Milan, Italy), a contact-only non-residual insecticide–acaricide based on plant-derived fatty acids, effective against the juvenile stages of several insects, including leafhoppers [32,33];
- *Beauveria bassiana* strain ATCC 74040 (Naturalis^®^, CBC Europe, Bergamo, Italy), an entomopathogenic fungus acting through cuticular penetration, active on both the larvae and adults of several pests [35,36,37];
- Kaolin (Surround^®^—Serbios s.r.l, Rovigo, Italy; Polvere di Roccia^®^, Biogard—CBC Europe, Bergamo, Italy), a 100% natural aluminum silicate clay formulation used as a physical particle film that interferes with host location and reduces leafhopper feeding behavior.

All products were applied at recommended label rates using a tower sprayer delivering 800–1000 L/ha; a tunnel sprayer was used only in 2021 (400 L/ha). Each year was treated as an independent field experiment, and results are therefore presented separately by season, reflecting the operational conditions of each trial. Applications were carried out following the mandatory application schedule rules established annually by the Plant Health Service of the Veneto Region for the control of Flavescence dorée. The spray program was scheduled to start upon the appearance of third-instar nymphs of *S. titanus*, as detected through regular monitoring. For each treatment, two consecutive applications of the same formulation spaced seven days apart were applied. Applications followed product-specific best practice recommendations; in particular, pyrethrin sprays were applied in the evening after 19:00 (around sunset), and the spray solution pH was adjusted to improve active ingredient stability, targeting pH 5.0–5.5. Assessments were performed immediately before the first insecticide application and at 7 days after the second application. Because assessments were carried out within the nymph-targeting window and shortly after the second application, adults were rare or absent at sampling dates and were not analyzed separately. For each replicate, 50 sampling units (basal leaves or suckers) were assessed along the two central rows, with basal leaves in 2021, 2022, 2024, and 2025 and basal suckers in 2023. Insects were counted directly in the field, recording for each unit the number of nymphs of *S. titanus*. Data were expressed as mean individuals per leaf (or per sucker in 2023). Insecticide efficacy was calculated using the Henderson & Tilton [38] formula.

### 2.2. Statistical Analyses

Statistical analyses were conducted separately for each experiment. Pre-treatment counts were used to check for baseline differences among treatments using a generalized linear mixed model with treatment as a fixed effect. Post-treatment counts (recorded approximately 7 days after the second application) were analyzed using generalized linear mixed models (PROC GLIMMIX, SAS ver. 9.4) assuming a Poisson or negative binomial distribution with a log link. Treatment was included as a fixed effect, and least-squares means were compared using Tukey’s test (α = 0.05). Model fit was assessed using residual diagnostics. Data are presented as means ± standard error, and different letters indicate significant differences among treatments.

## 3. Results

### 3.1. 2021

**Figure 1:**
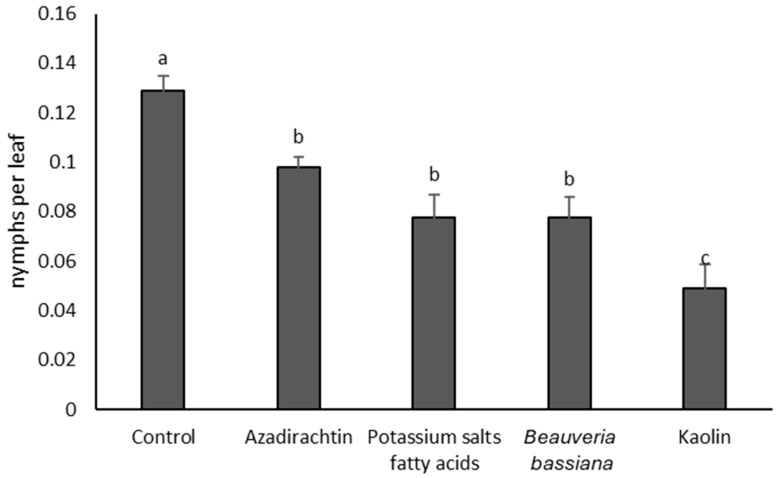
Densities (mean ± SE) of *Scaphoideus titanus* nymphs in the treatments compared in 2021. Different letters indicate significant differences among treatments (Tukey’s multiple comparison test on least-squares means, *p* < 0.05).

Before insecticide applications, *S. titanus* densities did not differ significantly among treatments (F = 0.04; df = 4, 10; *p* = 0.996). After insecticide applications, the treatment effect was significant (F = 9.62; df = 4, 10; *p* = 0.0002). *Scaphoideus titanus* densities were significantly higher in the untreated control than in treated plots (Figure 1). The use of kaolin produced the most marked decrease, whereas azadirachtin, *B. bassiana*, and potassium salts of fatty acids were associated with intermediate vector population levels (Figure 1).

### 3.2. 2022

**Figure 2:**
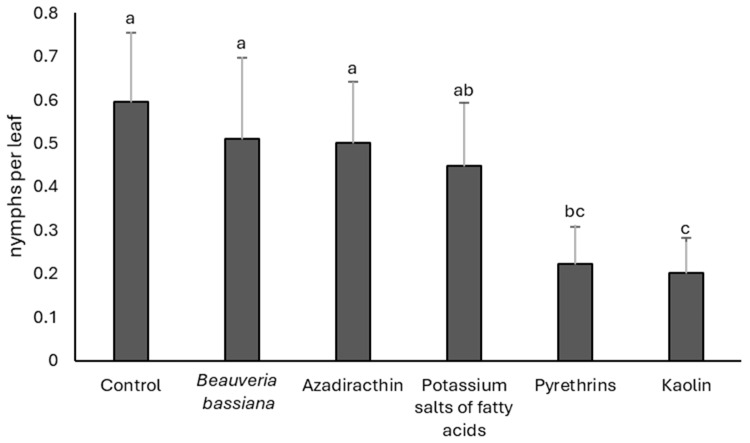
Densities (mean ± SE) of *Scaphoideus titanus* nymphs in the treatments compared in 2022. Different letters indicate significant differences among treatments (Tukey’s multiple comparison test on least-squares means, *p* < 0.05).

Initial populations were comparable among treatments (F = 0.24; df = 5, 15; *p* = 0.941). At the post-treatment assessment, insecticide applications had a significant effect on nymph densities (F = 5.67; df = 5, 15; *p* = 0.004). The untreated control, *B. bassiana*, and azadirachtin showed the highest population densities (Figure 2). Potassium salts of fatty acids resulted in intermediate densities, whereas pyrethrins and kaolin significantly reduced *S. titanus* compared with the untreated control.

### 3.3. 2023

**Figure 3:**
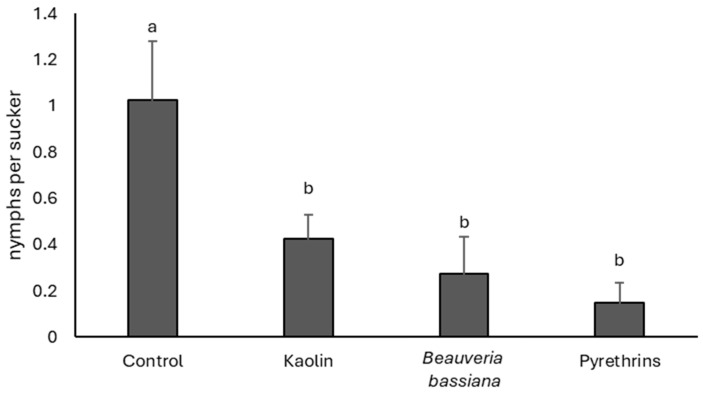
Densities (mean ± SE) of *Scaphoideus titanus* nymphs in the treatments compared in 2023. Different letters indicate significant differences among treatments (Tukey’s multiple comparison test on least-squares means, *p* < 0.05).

There were no differences in population densities among treatments before insecticide application (F = 1.95; df = 3, 9; *p* = 0.200). After insecticide application, the effect of treatment was significant (F = 6.55; df = 3, 9; *p* = 0.012). The highest *S. titanus* densities were found in the control plots (Figure 3), whereas the application of pyrethrins, kaolin, and *B. bassiana* was followed by a remarkable reduction in leafhopper numbers.

### 3.4. 2024

**Figure 4:**
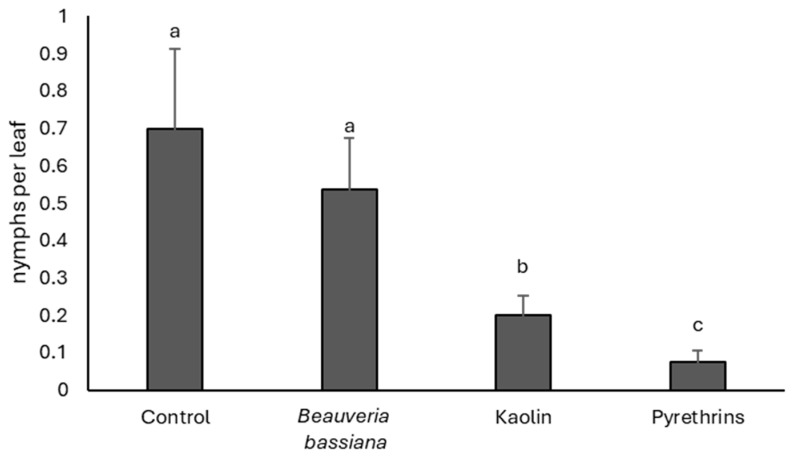
Densities (mean ± SE) of *Scaphoideus titanus* nymphs in the treatments compared in 2024. Different letters indicate significant differences among treatments (Tukey’s multiple comparison test on least-squares means, *p* < 0.05).

*Scaphoideus titanus* population densities in different treatments were similar before insecticide application (F = 0.56; df = 3, 9; *p* = 0.655), while they became significantly different after (F = 13.43; df = 3, 9; *p* = 0.002). The highest vector densities were associated with the untreated control and *B. bassiana* plots (Figure 4). Vector densities were significantly reduced using kaolin, but pyrethrins exerted the best control action.

### 3.5. 2025

**Figure 5:**
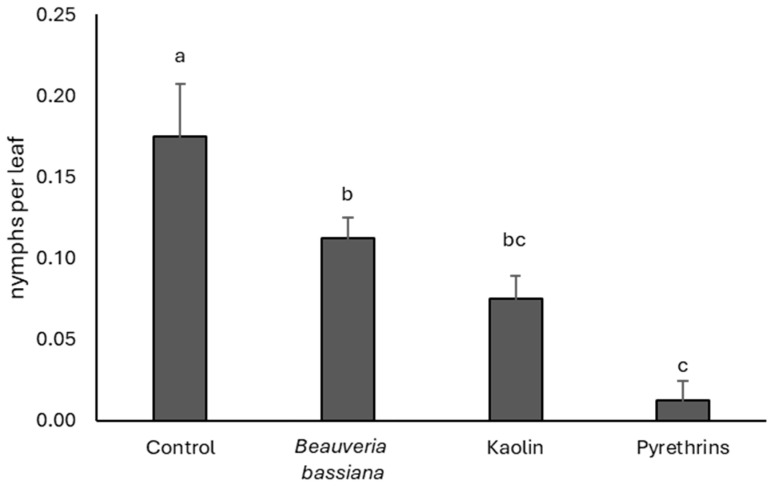
Densities (mean ± SE) of *Scaphoideus titanus* nymphs in the treatments compared in 2025. Different letters indicate significant differences among treatments (Tukey’s multiple comparison test on least-squares means, *p* < 0.05).

Differences among treatments were not significant before insecticide application (F = 0.3; df = 3, 9; *p* = 0.823), while they emerged thereafter (F = 5.61; df = 3, 9; *p* = 0.019). The highest *S. titanus* densities were recorded in the untreated control and the lowest were in the pyrethrin-treated plots (Figure 5). Kaolin did not differ from the latter treatment, while the use of *B. bassiana* gave intermediate numbers.

### 3.6. Evaluation of Insecticide Efficacy

Overall, the pattern observed across the 5-year study was consistent with the efficacy rates calculated using the Henderson & Tilton formula (Table 2). Over the years, pyrethrins were the most effective treatment, kaolin showed intermediate but stable efficacy, and azadirachtin and potassium salts of fatty acids were less effective, whereas *B. bassiana* effects were more variable.

## 4. Discussion

The natural insecticides tested against *S. titanus* nymphs exhibited clearly different levels of efficacy along the 5-year study. The absence of significant differences among treatments in the pre-insecticide sampling indicates that the variations observed in post-application assessments are attributable to the effects of the applied products rather than to initial imbalances in pest pressure.

Overall, pyrethrin-based insecticides proved to be the most effective and reliable tool, consistently resulting in the lowest post-treatment vector densities and in the highest Henderson–Tilton efficacy values, which frequently exceeded 80%, approached 90% in 2023–2024, and exceeded 90% in 2025. This aligns with previous reports on the rapid knock-down and good efficacy of natural pyrethrins [12]. However, it should be stressed that the persistence of pyrethrins is very limited, often lasting only a few hours under intense solar radiation [13,14], meaning that efficacy strongly depends on well-timed, repeated applications closely synchronized with vector phenology. Despite this limitation, the constant performance of the pyrethrin-based insecticides across years confirms that, at present, these insecticides represent the cornerstone of *S. titanus* control in organic systems, although attention must still be paid to potential impacts on beneficial arthropods [39,40,41,42].

Kaolin showed intermediate but comparatively stable efficacy across the trials. Its mode of action is based on physical–behavioral alterations rather than on specific biochemical targets: kaolin-based particle films interfere with visual cues, host finding, locomotion, and feeding, thereby reducing vector activity [17]. In our experiments, kaolin regularly reduced *S. titanus* densities to levels lower than or comparable to those obtained with other alternative products and provided intermediate Henderson–Tilton efficacy values, in agreement with previous studies reporting significant but moderate reductions in leafhopper populations [8,18,20]. Its performance was particularly satisfactory in warm, sunny seasons, supporting the hypothesis that kaolin films may be more disruptive under strong light conditions, whereas they are more vulnerable to wash-off under frequent rainfall. However, multi-year field trials conducted in Switzerland reported a much more variable and generally lower efficacy of kaolin, with a mean reduction of only 36.8% (range 0–88.9%) compared with 74.8% for natural pyrethrins; in that study, satisfactory control (>70%) was achieved only in a limited number of treatment events and mainly at low *S. titanus* densities, and kaolin efficacy decreased significantly as vector density increased, leading the authors to conclude that kaolin cannot be considered an efficient alternative to pyrethrins in mandatory control areas [10]. Although in our trials, kaolin was less effective than pyrethrins, its comparatively stable performance under our conditions and its agronomic benefits (such as the mitigation of thermal stress [19]) support its value within integrated management strategies against *S. titanus* and other leafhoppers. Even higher kaolin performance has been reported by Tacoli et al. [18], who reported kaolin efficacy comparable to pyrethrins when kaolin was applied twice versus two pyrethrin applications [8,18,20].

*Beauveria bassiana* showed moderate but highly variable efficacy across the years. Only in one experiment (2023) was it able to reduce *S. titanus* nymph densities on suckers to levels close to those obtained with pyrethrins and even lower than those recorded in the kaolin treatment, whereas in all other trials, its effect was more limited and consistently lower than that of pyrethrins and kaolin, as also reflected by the wide range of Henderson–Tilton values. This inconsistency can be attributed to the strong dependence of entomopathogenic fungi on microclimatic conditions: humidity, temperature, and UV radiation are known to affect spore germination and persistence on plant surfaces [26,27] and often constrain their performance in the field [35]. Overall, our results indicate that *B. bassiana* cannot be regarded as a decisive option for vector control in vineyards. Nonetheless, its potential endophytic activity [24,25] and well-documented safe profile [36] suggest that it may still play a role as a complementary tool within IPM programs, especially when applied repeatedly or under environmental conditions favorable to fungal infection.

Potassium salts of fatty acids provided limited-to-moderate control of *S. titanus* and, overall, were clearly less effective than pyrethrins and kaolin. In the seasons in which they were tested, nymph densities following application remained higher than those recorded in the pyrethrin and kaolin treatments, and the Henderson–Tilton efficacies were moderate. This outcome is consistent with previous studies on insecticidal soaps, which showed that formulations based on potassium salts of fatty acids can reduce populations of soft-bodied pests but often provide only moderate control under field or semi-field conditions [32,33,43,44]. Moreover, these products act mainly by disrupting the insect cuticle and cell membranes and require thorough, direct contact with the target insects while exhibiting negligible residual activity and a non-negligible risk of phytotoxicity on some plant species [45]. Under commercial vineyard conditions, where nymphs can shelter within the canopy and on the abaxial leaf surface, these constraints likely reduce the probability of lethal contact and help explain the relatively low efficacy observed in our trials. As a consequence, potassium salts of fatty acids do not appear suitable as stand-alone tools for *S. titanus* management under the commercial vineyard conditions tested here, but they may still contribute as complementary options in low-pressure situations and within diversified programs. Further field evaluations targeting early instars (N1–N2), when nymphs are more exposed and easier to reach by contact sprays, are warranted to better define their practical potential and optimal timing.

Azadirachtin showed the lowest efficacy among the tested products. Despite applications being carried out according to best practice recommendations (including evening sprays and conditioning of the spray solution, such as pH adjustment when appropriate), nymph densities remained similar to those of the untreated control, and reductions in *S. titanus* populations were generally modest compared with those obtained with pyrethrins and kaolin (see also the Henderson–Tilton values). Its mode of action, based on feeding inhibition and interference with molting, would theoretically favor activity on nymphs; however, under vineyard conditions, this translated into limited and inconsistent field performance. Overall, these results suggest that azadirachtin is unlikely to represent a core tool in organic vineyard programs; its practical value, if any, may be confined to complementary use within diversified strategies and should be clarified through targeted trials focusing on early instars (N1–N2) and optimized application conditions. This perspective remains consistent with its distinct physiological target and lack of cross-resistance with pyrethroids, as well as the broader interest in botanical insecticides for crop protection [46].

Efficacy estimates showed interannual variability. We did not perform a formal statistical comparison of efficacy between years because seasons differed in site conditions and operational settings (e.g., sprayer type/volume rate in 2021 and the sampling unit in 2023), and baseline nymph pressure and weather conditions varied across trials. Nevertheless, year-to-year differences in apparent efficacy are plausibly driven by variation in pre-treatment population density, rainfall and canopy microclimate affecting product persistence and coverage (e.g., wash-off of contact products and kaolin films), and meteorological conditions influencing fungal performance for *B. bassiana*.

The efficacy rates of natural insecticides calculated using the Henderson–Tilton formula are consistent with the pattern emerging from the post-treatment analyses. Pyrethrins consistently ranked as the most effective option, whereas kaolin provided intermediate but comparatively stable control over the years. *B. bassiana* and potassium salts of fatty acids achieved only moderate suppression and showed marked variability among trials, while azadirachtin displayed the lowest and most inconsistent efficacy. Although the performance of each product was affected to some extent by weather conditions and application timing, not all products were evaluated in every season. Nevertheless, when products were tested side-by-side, the relative efficacy pattern was consistent across the years, lending robustness to the conclusions and supporting the predictability of relative product performance under operational field conditions.

In addition to the biological aspects, the economic profile of the tested products is also relevant, as cost per hectare directly affects management decisions. In Italy, natural pyrethrins, although the most effective, cost between EUR 80 and EUR 150/ha per application, placing them in the medium-to-high-cost category. Kaolin, which showed intermediate but stable efficacy, is economically more sustainable, with relatively low costs per application (EUR 60–80/ha), even at the highest concentrations used. Azadirachtin is the most expensive option (EUR 150–200/ha/application), while *B. bassiana* is the least expensive (EUR 50–75/ha/application). Potassium salts of fatty acids are sold at prices that translate to about EUR 100–240/ha per single application, often exceeding the cost of both kaolin and pyrethrins. Considering the overall results, pyrethrin-based insecticides remain the products with the best cost–benefit ratio for achieving rapid reductions in vector populations, whereas kaolin represents a rational choice to complement control programs thanks to its moderate cost and stable performance. In contrast, azadirachtin, *B. bassiana*, and potassium salts of fatty acids show less favorable cost–efficacy profiles and should be employed only in specific, well-justified situations.

These findings support the implementation of diversified programs in which products are deployed according to their efficacy and cost–efficacy profiles and targeted to the appropriate phenological window rather than used as interchangeable alternatives or as single-product solutions; combining complementary tools and optimizing timing therefore emerges as a key requirement for sustainable control.

## 5. Conclusions

Across the five seasons, the relative ranking of products was broadly consistent; however, the sampling unit differed in 2023 (suckers) compared with the other seasons (leaves). This 5-year study highlights clear differences among the natural insecticides used, especially in organic viticulture for the control of *S. titanus*. Pyrethrins were confirmed as the most effective option for achieving a rapid and marked reduction in vector population densities and, at present, remain the reference tool for rapid knockdown in organic vineyards. Kaolin showed intermediate but comparatively stable levels of efficacy and emerged as the most reliable non-pyrethrin product, supporting its inclusion alongside pyrethrins in control programs aimed at reducing dependence on a single active ingredient. *B. bassiana* provided moderate but highly variable performance, likely influenced by environmental factors that limit spore persistence and infection. Potassium salts of fatty acids and azadirachtin consistently showed the lowest efficacy and therefore appear less suitable as core tools for vector management, although they may still be considered in specific, low-pressure situations or in localized interventions.

The observed efficacy ranking (pyrethrins > kaolin > *B. bassiana* > potassium salts of fatty acids > azadirachtin), supported by both statistical analyses and Henderson–Tilton calculations, provides a useful reference for designing *S. titanus* management strategies in organic vineyards. On this basis, a sustainable program should combine tools with complementary modes of action rather than rely on a single product. Pyrethrins should be reserved for the highest-risk periods, i.e., when monitoring indicates increasing nymphal densities or proximity to critical thresholds and a rapid knock-down of N2–N4 instars is required to prevent the build-up of *S. titanus* infective adults. Kaolin can be applied earlier and more continuously, from budbreak through to the main nymphal window, to hinder colonization and feeding by young instars while providing ancillary agronomic benefits. *B. bassiana* may be used as a complementary option during cooler and more humid periods, when environmental conditions favor fungal infection, or at relatively low pest densities. Azadirachtin and potassium salts of fatty acids, given their limited and inconsistent efficacy, should be confined to specific low-pressure scenarios or localized interventions. In the context of the limited introduction of new active ingredients and tangible resistance risk [16], defining these stage- and timing-specific roles and rotating or combining products appropriately is essential to support robust, diversified, and long-term control of the vector and, ultimately, Flavescence dorée.

## Figures and Tables

**Table 1:** Characteristics of products tested against *Scaphoideus titanus* in organic vineyards. In different experiments, products were applied according to label rates and sprayer features.

Product (Trade Name)	Active Ingredient/Strain	Formulation	Maximum Application Rates
Biopiren^®^ Plus	Natural pyrethrins	EC (emulsifiable concentrate)	2.4 L product/ha 160 mL/hL
Oikos^®^	Azadirachtin A (26 g/L)	EC/liquid formulation	1.5 L product/ha 180 mL/hL
Flipper^®^	Potassium salts of fatty acids (479.8 g/L)	Liquid concentrate	20 L product/ha
Naturalis^®^	*Beauveria bassiana* strain ATCC 74040	Oil dispersion (OD)/suspension	1.5 L product/ha
Surround^®^ Polvere di Roccia^®^	Kaolin (100% aluminum silicate)	Wettable powder	20–30 kg product/ha

**Table 2:** Henderson–Tilton corrected efficacy (%) of the tested products against *Scaphoideus titanus*.

Product	Mean	2021	2022	2023	2024	2025
Natural pyrethrins	83	-	57	90	90	93
Kaolin	58	63	63	55	68	42
*Beauveria bassiana*	37	38	13	77	21	36
Azadirachtin	27	42	12	-	-	-
Potassium salts	32	44	20	-	-	-

## Data Availability

The original contributions presented in this study are included in the article. Further inquiries can be directed to the corresponding authors.
